# Lvr, a Signaling System That Controls Global Gene Regulation and Virulence in Pathogenic *Leptospira*

**DOI:** 10.3389/fcimb.2018.00045

**Published:** 2018-02-23

**Authors:** Haritha Adhikarla, Elsio A. Wunder, Ariel E. Mechaly, Sameet Mehta, Zheng Wang, Luciane Santos, Vimla Bisht, Peter Diggle, Gerald Murray, Ben Adler, Francesc Lopez, Jeffrey P. Townsend, Eduardo Groisman, Mathieu Picardeau, Alejandro Buschiazzo, Albert I. Ko

**Affiliations:** ^1^Department of Epidemiology of Microbial Diseases, Yale School of Public Health, New Haven, CT, United States; ^2^Laboratory of Molecular & Structural Microbiology, Institut Pasteur de Montevideo, Montevideo, Uruguay; ^3^Yale Centre for Genome Analysis, West Haven, CT, United States; ^4^Department of Biostatistics, Yale School of Public Health, New Haven, CT, United States; ^5^Gonçalo Moniz Research Center, Oswaldo Cruz Foundation, Salvador, Brazil; ^6^Lancaster Medical School, Lancaster, United Kingdom; ^7^Department of Microbiology, Biomedicine Discovery Institute, Monash University, Clayton, VIC, Australia; ^8^Australian Research Council Centre of Excellence in Structural and Functional Microbial Genomics, Monash University, Clayton, VIC, Australia; ^9^Department of Microbial Pathogenesis, Yale University School of Medicine, New Haven, CT, United States; ^10^Institut Pasteur, “Biology of Spirochetes” Unit, Paris, France; ^11^Department of Microbiology, Institut Pasteur, Paris, France

**Keywords:** Leptospira, pathogenic, branched signaling, two-component system, hybrid histidine kinase, hybrid response regulator, virulence, gene duplication

## Abstract

Leptospirosis is an emerging zoonotic disease with more than 1 million cases annually. Currently there is lack of evidence for signaling pathways involved during the infection process of *Leptospira*. In our comprehensive genomic analysis of 20 *Leptospira* spp. we identified seven pathogen-specific Two-Component System (TCS) proteins. Disruption of two these TCS genes in pathogenic *Leptospira* strain resulted in loss-of-virulence in a hamster model of leptospirosis. Corresponding genes *lvrA* and *lvrB (leptospira virulence regulator*) are juxtaposed in an operon and are predicted to encode a hybrid histidine kinase and a hybrid response regulator, respectively. Transcriptome analysis of *lvr* mutant strains with disruption of one (*lvrB*) or both genes (*lvrA/B*) revealed global transcriptional regulation of 850 differentially expressed genes. Phosphotransfer assays demonstrated that LvrA phosphorylates LvrB and predicted further signaling downstream to one or more DNA-binding response regulators, suggesting that it is a branched pathway. Phylogenetic analyses indicated that *lvrA* and *lvrB* evolved independently within different ecological lineages in *Leptospira* via gene duplication. This study uncovers a novel-signaling pathway that regulates virulence in pathogenic *Leptospira* (Lvr), providing a framework to understand the molecular bases of regulation in this life-threatening bacterium.

## Introduction

Leptospirosis is a zoonotic disease caused by pathogenic species of the genus *Leptospira*. Infection with this pathogen results in more than one million human cases a year with a fatality ratio frequently exceeding 10% (Bharti et al., 2003; Costa et al., 2015a). The life cycle of pathogenic *Leptospira* is complex, including asymptomatic reservoir and susceptible hosts (Ko et al., 2009). Large spectrum of mammalian hosts including rodents (the Norway rat, *Rattus norvegicus* and/or the black rat, *Rattus rattus*), live stock, dogs, and horses harbor and shed the pathogenic leptospires from their renal tubules into the environment (Ko et al., 1999; Costa et al., 2015b). Transmission of *Leptospira* to humans is due to exposure of risk groups to animal reservoirs or contaminated environments (Reis et al., 2008; Costa et al., 2015b). Therefore, pathogenic *Leptospira* must adapt rapidly to the versatile ecological niches encountered during its lifecycle. The *Leptospira* genomes encode an array of serine-threonine protein kinases (STPKs), extra cytoplasmic function (ECF) sigma factors, and two-component signal transduction systems (TCSs), which might enable *Leptospira* to traverse the diverse environmental stimuli experienced during the infection (Nascimento et al., 2004; Fouts et al., 2016). However, TCSs in *Leptospira* have been implicated only in heme metabolism (Louvel et al., 2008; Morero et al., 2014) and chemotaxis (Lambert et al., 2015) but not in virulence regulation. Here we provide a singular example of a virulence governing, non-classical TCS in pathogenic *Leptospira*.

The *Leptospira* genomes encode a substantially higher number of TCS genes (>50) compared to other spirochetes such as *Borrelia spp*. (<10) and *Treponema spp*. (<20). A pan-genus genomic analysis of globally representative 20 *Leptospira* species revealed that a high percentage of TCS genes (60%) in *Leptospira* encode non-classical TCSs (Fouts et al., 2016) and most of them are designated hybrid response regulators (HRR). Non-classical TCSs do not comply with a linear phosphate flow from sensor histidine kinases (HKs) to cognate response regulators (RRs), as observed in classical TCSs (Mascher et al., 2006).

HRRs comprise an N-terminal domain harboring the ultra-conserved aspartate residue that receives the phosphoryl group from upstream TCS partners (denominated receiver domain or REC), followed by C-terminal modules typical of HKs within the same polypeptide (Wuichet et al., 2010). HRRs remain largely unexplored with very few reports in endo-symbiotic bacteria and plant-associated bacteria (Wojnowska et al., 2013; Kaczmarczyk et al., 2015).

In this study, we report a novel hybrid histidine kinase / hybrid response regulator pair LvrAB (***L****eptospira* **v**irulence **r**egulator), specific to pathogenic *Leptospira*. Our findings determine the global regulatory role of *lvr* genes with a special emphasis on their role in virulence. Moreover, our study suggests that LvrA/B operate through a branched signaling pathway, predicting that a specific downstream DNA-binding response regulator(s) functions as final effector(s).

## Materials and methods

### Bacterial cultures and growth conditions

*Leptospira interrogans* serovar Manilae strain L495 WT (WT) and mutant strains (lvrA/B, lvrA/B II, lvrB, lic13192, lic13087, lic11713) were cultured in Ellinghausen-McCullough-Johnson-Harris liquid medium (EMJH) (Johnson and Harris, 1967) supplemented with 1% rabbit serum (Sigma-Aldrich) at 30°C with shaking (100 rpm). Leptospires were enumerated by dark-field microscopy in a Petroff-Hausser chamber (Thermo Fisher Scientific, Waltham, MA, USA). When appropriate, spectinomycin or kanamycin was added to the culture medium at the final concentration of 40 μg/ml.

Motility of the WT and mutant Leptospira (lvrA/B, lvrA/B II, lvrB) was evaluated by inoculating 105 leptospires onto semisolid EMJH medium containing 0.5% agar (DifcoTM Noble Agar, BD Biosciences, NJ, USA). The plates were incubated at 30°C without shaking and the diameters of the growth zones were measured after 14 days. Assays were repeated in triplicate.

### Random mutagenesis

A plasmid vector pSC189 containing both the hyperactive transposase C9 and transposon terminal inverted repeats flanking a kanamycin resistance gene (Bourhy et al., 2005) was used to deliver Himar1 in the L. interrogans serovar Manilae strain L495 genome and random mutants were selected. Representative Kmr clones were further tested by PCR for the identification of the insertion site of the Himar1 transposon in the genome using primers flanking the site of insertion, followed by DNA Sanger sequencing (Table S2).

### Site-directed mutagenesis of Lvr proteins

LvrA and LvrB coding genes were amplified from *L. interrogans* serovar Copenhageni Fiocruz L1-130 genomic DNA, and cloned into a modified version of the pQE80L (Qiagen Kit, Germantown, MD, USA) plasmid using the Restriction Free (RF) method (Van Den Ent and Lowe, 2006). This plasmid (pQE80L-TEV) includes a TEV cleavage site and a GSGS linker after the N-terminal His-tag. RF cloning, using the appropriate mutagenic oligonucleotides also generated point mutants. Full-length LvrA or LvrB protein variants were expressed in *Escherichia coli* (TOP10F′) and purified by affinity chromatography using standard procedures.

### Virulence test

*In vivo* screening was performed to test the virulence potential of Leptospira WT and mutant strains (lvrA/B, lvrA/B II, lvrB, lic13192, lic13087, lic11713). Golden Syrian male hamsters were challenged via conjunctival route with doses of 5 × 106, 107, or 108 leptospires in 10 μl of EMJH, as described previously (Wunder et al., 2016). Animals were monitored for clinical signs of disease up to 21 days post-challenge. Sick animals were immediately euthanized by inhalation of CO2, and death was used as primary outcome. Treatment effects (mutations in L. interrogans Manilae strain) and day effects were estimated based on proportional hazards model.

For studying expression pattern of *lvr* genes *in vivo*, golden Syrian male hamsters (*n* = 2) were challenged with *Leptospira* WT (10^8^ leptospires in 1 mL of EMJH) via intraperitoneal (IP) route as described previously (Wunder et al., 2016). Animals were monitored for clinical signs of disease up to 21 days post-challenge and sick animals were immediately euthanized by inhalation of CO_2_. Blood samples were collected by retro-orbital bleeding procedure from these hamsters at defined intervals (1 day, 3 days, 5 days, and 7 days) and stored in TRIzol (Invitrogen^TM^, Thermo Fisher Scientific, Waltham, MA, USA) at −80°C until further use.

### Ethics statement

All animal protocols and work were approved and conducted under the guidelines of the Yale Institutional Animal Care and Use Committee (IACUC), under approved protocol #2014–11424. The Yale IACUC strictly adheres to all Federal and State regulations, including the Animal Welfare Act, those specified by Public Health Service, and the US Department of Agriculture, and uses the *US Government Principles for the Utilization and Care of Vertebrate Animals Used in Testing, Research, and Training* as a guide for all animal studies.

### RNA extraction, library preparation, and RNA-sequencing

*Leptospira* cells of WT and mutant strains (*lvrA/B* and *lvrB*) were cultured in EMJH (Johnson and Harris, 1967) supplemented with 1% rabbit serum (Sigma-Aldrich, St. Louis, MO, USA) at 30°C with shaking (100 rpm) and subsequently harvested at late-log phase by centrifugation at 3,200 *g*. RNA was extracted for two biological replicates using the TRIzol method (Invitrogen^TM^, Thermo Fisher Scientific, Waltham, MA, USA).

Six strand-specific sequencing libraries, two for each strain, were produced from total RNA. The libraries were run on HiSeq 2000, generating approximately between 21 and 35 million pair-end reads of 76 bp each. Adapter sequences, empty reads, and low-quality sequences were removed. The first and the last nucleotides with quality scores below 20 for each read were trimmed to remove low quality bases using in-house scripts. After trimming, reads shorter than 45 bp were also discarded. Trimmed reads were mapped to *L. interrogans* serovar Manilae L495 with a known transcriptome index (http://www.genoscope.cns.fr/) using Tophat v2.0.11 (Trapnell et al., 2009). Only reads that mapped to a single unique location in the genome with a maximum of two mismatches in the anchor region of the spliced alignment were reported in these results. To obtain a tally of the number of the reads that overlapped the exons of a gene, we analyzed the aligned reads with HTSeq v0.6.1p1 (Anders et al., 2015) (http://www-huber.embl.de/users/anders/HTSeq/doc/). Differential expression analysis was performed with DESeq2 (Love et al., 2014). *P*-values were corrected for multiple testing with Benjamini-Hochberg correction.

We identified 850 genes with at least 2 log_2_-fold changes and with a significance of *p*-adjusted < 0.05 in each comparison group. Hierarchical clustering was performed with 850 genes exhibiting significant changes across all conditions and a heat map was generated with the regularized-logarithm transformation of the data (Schmittgen and Livak, 2008). PCA plots showed samples clustering by treatment group.

### RNA seq data analyses

The sequences were aligned to the *L. interrogans* serovar Manilae L495 obtained from http://www.genoscope.cns.fr. The reference genome was indexed using the bowtie2-index. The reads were pre-processed to remove the first and last base, and filtered for quality using scripts written in PERL. The reads were aligned to the reference genome using Tophat (Trapnell et al., 2009). The aligned bam files were processed using htseq-count (Anders et al., 2015), and the raw counts were processed using DESeq2 package in R (Love et al., 2014). The downstream analysis and plotting was done using home brew scripts written in R (R Archive Network). R package pheatmap was employed for the heatmap where the values were scaled according to row to depict how the gene expression for the specific gene changed across the conditions.

### Whole genome sequencing

*L. interrogans* serovar Manilae mutant strains (*lvrA/B* and *lvrB)* were cultured in Ellinghausen-McCullough-Johnson-Harris liquid medium (EMJH) (Johnson and Harris, 1967) supplemented with 1% rabbit serum (Sigma-Aldrich, St. Louis, MO, USA) at 30°C with shaking (100 rpm). DNA was then extracted from late-log phase cultures by Maxwell 16 DNA purification kit (Promega, Fitchburg, WI, USA). The quality and concentration of DNA was measured by NanoDrop^TM^ 2000 spectrophotometer (Thermo Fisher Scientific, Waltham, MA, USA) and by fluorometric assay using the Quanti-iT PicoGreen dsDNA assay kit (Invitrogen^TM^, Thermo Fisher Scientific, Waltham, MA, USA).

The genomes of the isolates *lvrA/B* and *lvrB* were sequenced at the Yale Center for Genome Analysis (YCGA) using the Illumina HiSeq2000 sequencing system. The sequenced reads were mapped to *L. interrogans* serovar Manilae L495 genome (http://www.genoscope.cns.fr/) and Geneious software package was employed for variant calling. In order to confirm a variant call, a minimum of 75% of the sequencing reads should support the call.

### Quantitative reverse transcription PCR (RT-qPCR) for target gene identification and RNA seq data validation

*Leptospira* WT and *lvr* mutants were cultured till mid-log and late-log phases. RNA was extracted for two biological replicates using the TRIzol method (Invitrogen^TM^, Thermo Fisher Scientific, Waltham, MA, USA). For studying expression of *lvr* genes *in vivo*, total RNA was extracted from hamster blood samples (*n* = 2) stored in TRIzol, as per the manufacturer's instructions (Invitrogen^TM^, Thermo Fisher Scientific, Waltham, MA, USA).

Ambion® TURBO DNA-free™ DNase treatment kit (Applied Biosystems Inc, Foster City, CA, USA) was employed to remove contaminating DNA from RNA preparations. The concentration of RNA was determined using a NanoDrop^TM^ 2000 spectrophotometer system (Thermo Fisher Scientific, Waltham, MA, USA). The High capacity cDNA reverse transcription kit (Applied Biosystems Inc, Foster City, CA, USA) was employed for conversion of total RNA to single-stranded cDNA. The primers designed for target gene identification and RNA-seq data validation are listed in Table S2.

The qPCR was carried out on 7500 fast real-time PCR (Applied Biosystems Inc, Foster City, CA, USA) using iQ^TM^ SYBR^R^ Green supermix (Bio-rad, Hercules, CA, USA) according to manufacturer's instructions. The thermal cycling conditions used in the qPCR were 95°C for 3 min, followed by 40 cycles of 95°C for 5 s and 60°C for 1 min. The specificity of the SYBR green PCR signal was confirmed by melt curve analysis. In RT-qPCR experiments, *flaA* gene (flagellar apparatus gene) was used as an endogenous control and *L. interrogans* WT was employed as reference strain. A relative quantification analysis was performed using the comparative CT method, and the relative gene expression was calculated by using the 2^−Δ*ΔCt*^ method (Schmittgen and Livak, 2008).

### Molecular evolution analyses

Multiple BLAST searches were performed against the NCBI database for homologs of *Lvr* in other bacterial genomes. Two Lvr homologs in *Mycobacterium* were employed to root the phylogeny. A set of 67 unique amino acid sequences was assembled and they were aligned using SATé-II (Liu et al., 2012) with MAFFT as aligner, MUSCLE as merger, and RAxML as tree estimator under WAG model. The robustness of branching topologies was estimated with 1,000 maximum likelihood searches of bootstrapped sequence data using PhyML (Guindon et al., 2010) under the WAG model. Robustness of the topology was further confirmed with Bayesian analyses using MrBayes 3.2 (Ronquist et al., 2012). Bayesian phylogenetic analyses were performed using the Metropolis-coupled Markov chain Monte Carlo method (MCMCMC) under a mixed amino acid model by running for chains with 10,000,000 generations. Trees were sampled every 1000th generation, and first 2,000 trees were discarded before computing a consensus tree. Branches with bootstrap proportions (BP) higher than 80% or posterior probability (PP) higher than 0.98 were considered significantly supported.

A total of 67 sequences with 1,199 aligned positions were included in the phylogenetic analyses. The tree was rooted with orthologs of *Mycobacterium* (*M. gastri* and *M. kansasii* genes, with 42% identity). There were no significant conflicts in topology of the gene trees as inferred by maximum likelihood analyses using PhyML and Bayesian analyses; branch support was significant (bootstrap >80% and posterior probabilities ≥0.98). A consensus tree based on the last 8,001 trees sampled per 1,000 generations in Bayesian analyses featured a Bayesian posterior probability of ≥98%.

### Phosphotransfer assays

Purified Lvr proteins (1 mg/mL) in desalting buffer (25 mM Tris-HCl, 250 mM NaCl, 10 mM MgCl2, pH 8.5) were incubated with 1 mM ATP and 10 mCi of [γ^33^P] ATP (3000 Ci/mmol, ARC) at room temperature for 30 mins. Phosphotransfer reactions were stopped by mixing the samples with Laemmli buffer. Samples were then subjected to gel electrophoresis (SDS-PAGE), and visualized by autoradiography (Hyperfilm ECL) after 1–3 days of exposure. Densitometry analysis was performed using the software ImageJ.

### Statistical analysis

GraphPad Prism (Prism Mac 5.0) was employed for statistical analysis of *in vivo* and motility data. Fisher's exact test was used to calculate *p*-values for difference in mutant infections clearance with 2 × 2 contingency table. The Benjamini-Hochberg FDR method was used to adjust the *p*-values for multiple testing of RNA-seq data (Noble, 2009). Spearman and Pearson's *R*-value determined similarities between RNA replicates.

### Data availability

RNA-Seq reads are available in the NCBI Sequence Read Archive, under accession numbers *L. interrogans* Manilae wild type (I: GSM2085874, II: GSM2085875), *lvrA/B* mutant (I: GSM2085878, II: GSM2085879), *lvrB* mutant (I: GSM2085876, II: GSM2085877).

The genome sequencing data for *lvrA/B* and *lvrB* mutants have been deposited in NCBI under accession numbers SRR5956150 and SRR5956154 respectively.

## Results and discussion

### Identification of a TCS governing virulence in *Leptospira*

Our comparative genomic analysis of 20 *Leptospira* species (Fouts et al., 2016) led us to the identification of seven genes encoding TCS proteins in all the pathogenic *Leptospira* species but distinctly absent from saprophytic spp. Therefore, we speculated the role of these seven signaling proteins in pathogenic mechanisms and/or virulence modulation. As a further step to identify the regulatory role of these putative signaling proteins, we screened our *Mariner* transposon based mutant library in pathogenic *L. interrogans* serovar Manilae. In our screening of mutant library, we identified disruptions in four pathogen-specific TCS genes (Tables S1, S2). Two of the mutants had insertions in a pathogen-conserved locus encoding a hybrid HK (gene *lic11709*) and a hybrid RR (gene *lic11708*). The other two mutant strains were found to have insertions in a classical HK gene (*lic13087)* and a hybrid HK gene (*lic13192*) (Table S1).

**Figure 1:**
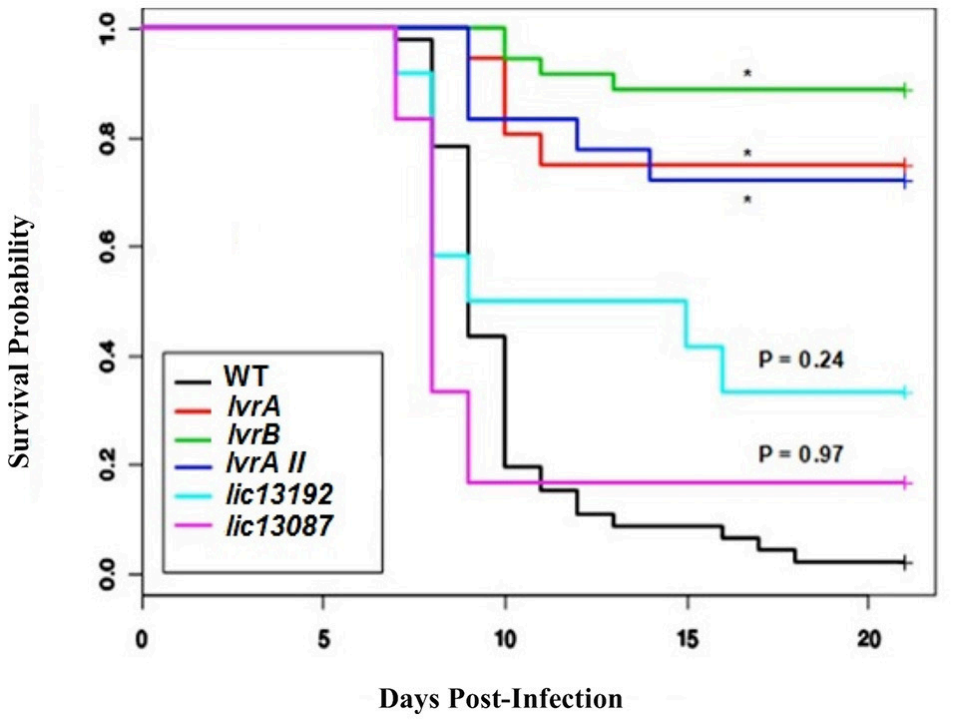
*In vivo* screening of selected *Leptospira* TCS mutants. Golden Syrian male hamsters were challenged with wild type, *lvrA/B* (M1529)*, lvrA/BII* (M1529 II)*, lvrB* (M1419)*, lic13192* (M480), and *lic13087* (M854) mutants of *L. interrogans* Manilae L495 sp via conjunctival route in doses of 5 × 10^6^, 10^7^, and 10^8^ leptospires. Animals were monitored for 21 days post-challenge with death as a primary outcome. The survival probability plot was based on a proportional hazards model. Treatment effects (mutations in *L. interrogans* Manilae strain) and day effects were estimated based on this model and *P* values were calculated. ^*^P < 0.0001.

Subsequently, we aimed to determine the ability of the above TCS mutants to cause infection in a hamster model. A significant loss of virulence (*p* < 0.05) was observed in the survival curves of hamsters infected with *lic11708* and *lic11709* mutants when compared to wild-type *L. interrogans* (WT) (Figure 1, Tables S3, S4). By contrast, *lic13192* and *lic13087* mutants did not show significant decrease in hamster mortality (*p* > 0.05; Figure 1). All four strains showed similar bacterial growth in EMJH medium (data not shown) indicative that virulence defects of the *lic11708* and *lic11709* mutants are due to their altered virulence potential in a mammalian host. Taking into account the *in vivo* virulence attenuation phenotype, this TCS pair is designated as Lvr, ***L****eptospira* **v**irulence **r**egulator system, comprising proteins LvrA (*lic11709*) and LvrB (*lic11708*).

Whole genome sequencing of *lvrA* (*lic11709*) and *lvrB* (*lic11708*) mutants revealed single transposon insertion events in corresponding *lvr* genes respectively. Thus, we confirmed that the attenuated phenotype exhibited by these mutant strains is attributable to specific insertions in *lvr* genes but not due to any off-target mutations. Genome sequencing results therefore compensated for our unsuccessful attempts to complement the *lvr* mutant strains with corresponding wild-type genes. Furthermore, an independent transposon mutant *lvrA_*II was additionally identified in *lvrA* gene locus (Table S1) and it also displayed an *in vivo* attenuated phenotype (Figure 1). Taken together, the evidence provided here strongly supports a virulence modulation function of the Lvr signaling system in pathogenic *Leptospira* spp.

### Lvr constitutes an unusual “hybrid histidine kinase/hybrid response regulator” pair

**Figure 2:**
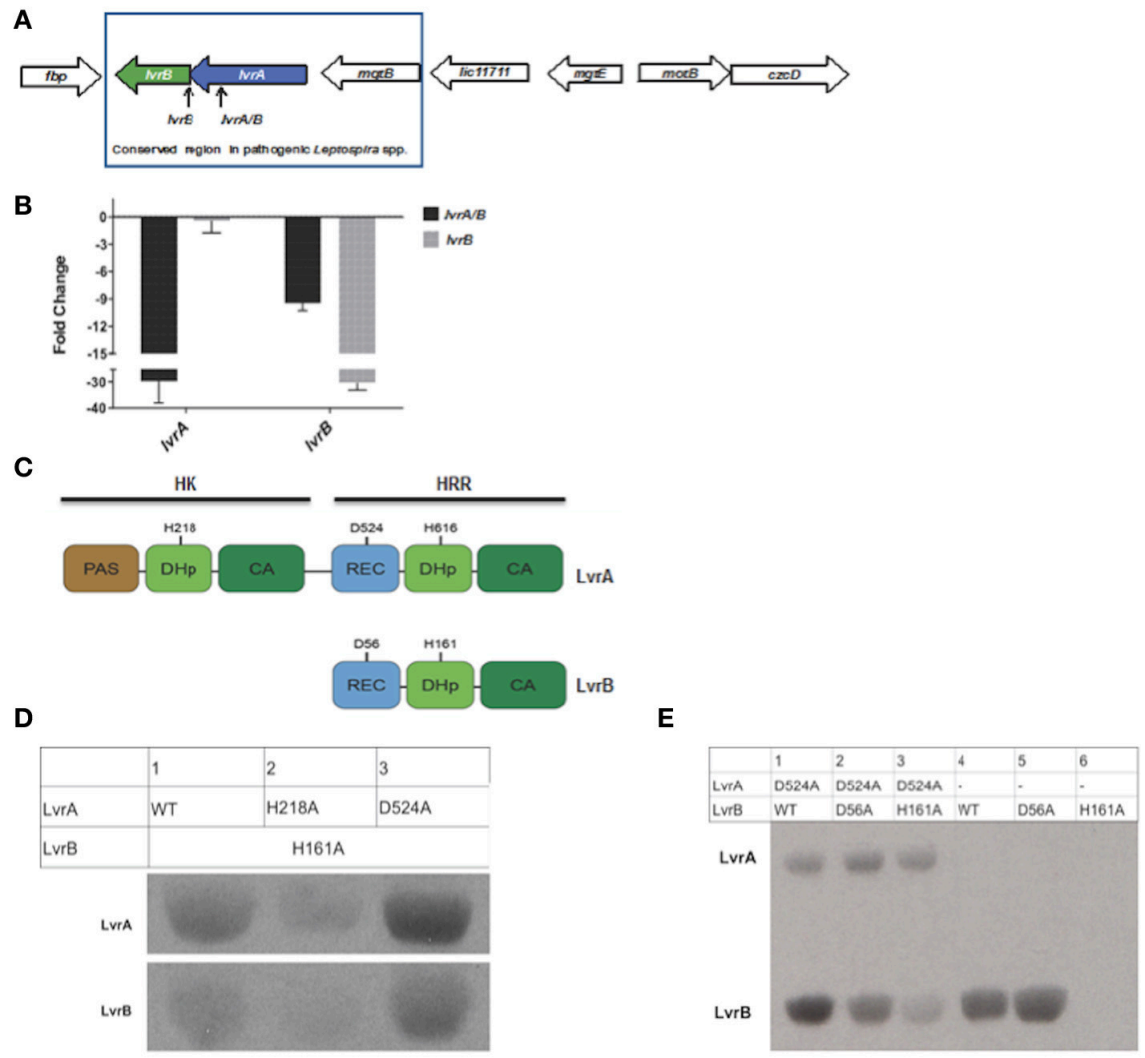
Gene arrangement and domain organization of Lvr dyad in pathogenic *Leptospira* spp. **(A)** This figure is a schematic representation of the Lvr loci in pathogenic Leptospira. Arrow length is proportional to the gene length. *lvrA* (blue): Putative hybrid histidine kinase-response regulator gene and *lvrB* (green): Putative hybrid response regulator gene in *L. interrogans* serovar Manilae strain L495. Himar1 insertion site in *lvrA* gene (*lvrA/B* mutant) and in intergenic region between *lvrA* and *lvrB* (*lvrB* mutant) has been indicated. **(B)** *In vitro* gene expression of *lvrA* and *lvrB* by RT-qPCR **(C)** Domain organization of Lvr proteins; signature segments of kinase and receiver domains were identified by multiple sequence alignment and indicated. Conserved histidine and aspartate residues as putative phosphorylation sites are denoted for each protein. REC, receiver domain; PAS, Per, Arnt, Sim domains; DhP, Dimerization histidine phosphotransfer; CA, catalytic and ATP-binding domain. **(D)** Phosphotransfer from LvrA to LvrB. An LvrB mutant devoid of autokinase activity (LvrB_H161A) was incubated with [Y-33P] ATP for 30 min with LvrA wild type, LvrA_H218A or LvrA_D524A (as labeled in each lane). Reaction products were separated by SDS-PAGE and visualized using autoradiography. **(E)** Wild-type LvrB and the point mutants D56A and H161A were incubated with [Y-33P] ATP and MgCl2 for 30 min, in presence (lanes 1 to 3) or absence (lanes 4 to 6) of LvrA_D524A (as labeled in each lane). Reaction products were separated by SDS-PAGE and visualized using autoradiography.

Pathogenic *Leptospira* spp. uniquely harbor the genomic region encompassing two genes, *lvrA* and *lvrB*, which appear to be part of the same operon (Figure 2A). Consistent with this notion, the *lvrA* and *lvrB* transcripts were 29- and 9- fold lower than the wild type strain in an *lvr* mutant with a transposon insertion in the *lvrA* gene (Figure 2B). Hereafter, *lvrA* strain is thus named as *lvrA/B* mutant. By contrast, the *lvrB* mutant with a transposon insertion in the intergenic region between *lvrB* and *lvrA* resulted in a 30-fold downregulation of the *lvrB* transcript only, with *lvrA* expression unchanged (Figure 2B).

The *lvr* genes encode for TCS proteins with unusual domain architectures (Goulian, 2010). The 832 amino acid long LvrA is a hybrid HK (HHK) protein, with a canonical N-terminal HK region and a C-terminal RR region within the same polypeptide (Figure 2C). The HK region comprises a PAS sensory domain (spanning residues 11–141), a Dimerization and Histidine phosphotransfer (DHp) domain (206–270) and a Catalytic and ATP-binding (CA) domain (residues 325–427). The RR region that follows is unusual in that it comprises a typical REC domain (residues 478–583) that is followed by an effector domain resembling a HK on its own (Figure 2B), comprising a DHp domain (residues 604–670) and CA domain (residues 720–823). Each of the two HK portions in LvrA includes the histidine residue within the conserved H-box motifs, which could thus be phosphorylated during signaling, at positions 218 and 616. A potentially phosphorylatable aspartate residue within LvrA's RR domain is found at position 524 (Figure 2B).

The 382 amino acid long LvrB protein has a domain organization corresponding to a HRR, similar to the C-terminal half of LvrA (Figure 2C). LvrB thus comprises a REC domain (residues 7–120), including conserved Asp56 at the putative phosphorylation site, upstream of an HK core. The latter displays a DHp domain (residues 149–213) harboring a phosphorylatable His at position 161, and a CA domain (residues 264–374; Figure 2C). By using an *in silico* approach [http://topcons.cbr.su.se/] we predicted the cytoplasmic localization of Lvr proteins. Also absence of transmembrane regions in LvrA or LvrB proteins confirms their cytoplasmic localization. Interestingly, DNA binding domains are equally absent in both Lvr proteins, instead their corresponding REC domains are connected to enzymatic (HK) effector domains.

We hypothesized that the LvrA and LvrB proteins are cognate partners within a phosphotransfer cascade pathway, because the *lvrA* and *lvrB* genes are co-transcribed as part of an operon. We tested this hypothesis by quantifying phosphotransferase activity *in vitro* using wild type (LvrB_wt) and mutant versions of full-length LvrA and LvrB proteins, purified as soluble recombinant species from *E. coli*. We employed autokinase-defective mutant LvrB_H161 as a negative control in which the phosphorylatable His161 was substituted by alanine (LvrB_H161A). Incubation of LvrB_H161A with variants of LvrA (wild-type, LvrA_H218A, and LvrA_D524A) in the presence of [γ^33^P] ATP, showed that LvrA catalyzes the transfer of its His-bonded phosphoryl group to LvrB (Figure 2D). To study the effect of Asp56 phosphorylation on the autokinase activity of LvrB, we performed autophosphorylation assays in the presence or absence of LvrA_D524A (Figure 2E). The autokinase activity of wild-type LvrB significantly increased in presence of its partner, respect to the basal level of activity detected in its absence. In contrast, the activity of the LvrB_D56A mutant was not affected, displaying basal activity levels in both cases. Taken together, these results suggest that phosphotransfer occurs from LvrA His218 to the receiver Asp56 within LvrB, indeed supporting the hypothesis that LvrA and LvrB are part of the same signaling pathway.

### LvrAB modulates global transcriptional regulation

**Figure 3:**
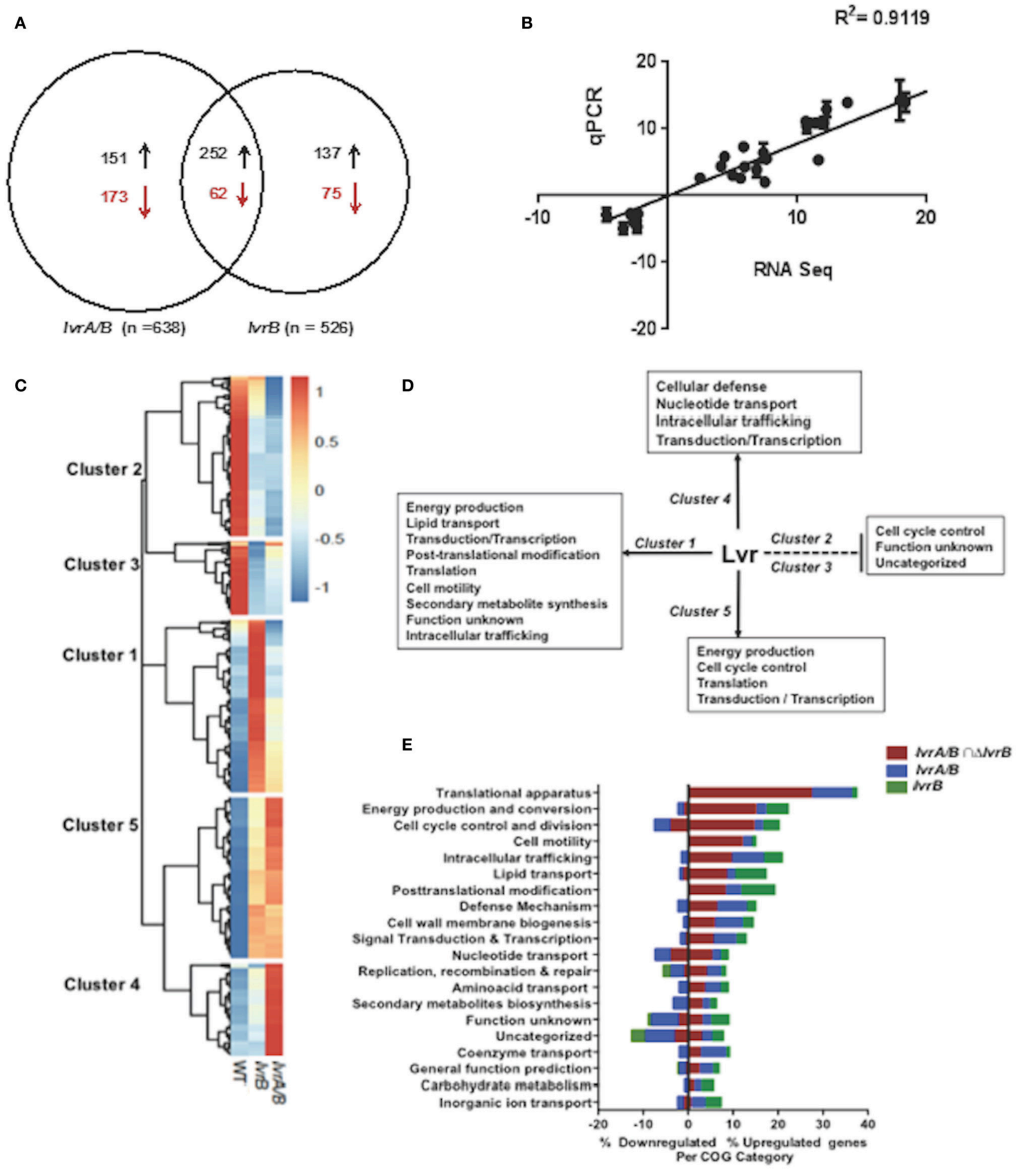
Global transcriptional changes in *lvr* mutants. **(A)** Venn diagram depicting the number of differentially expressed genes in *lvr* mutants, *lvrA/B* (M1529) and *lvrB* (M1419) with ± log 2-fold change cut-off and *P* ≤ 0.05. **(B)** Validation of RNA-Seq Analysis was performed by RT-qPCR and correlation coefficient has been indicated. **(C)** Heatmap depicting clusters of differentially expressed genes in *lvr* mutants when compared to *L. interrogans* Manilae L495 WT. Computationally we identified five arbitrary clusters that are marked in the heat map. **(D)** Lvr regulatory functions inferred from transcriptome analysis of *lvr* mutants, *lvrA/B* (M1529) and *lvrB* (M1419). Solid and dashed lines depict positive regulation and negative regulation, respectively. Inferences are based on relative abundance of COG categories (>5%) across each cluster. **(E)** Functional categorization of upregulated and downregulated genes in *lvr* mutants, *lvrA/B* (M1529) and *lvrB* (M1419) during late-log phase of growth at 30°C. Percent distribution is calculated for the total number of differentially expressed genes (according to the RNA-Seq analysis; log 2-fold change, *P* < 0.05) in each COG category.

To uncover the physiologic role of the Lvr system, we examined the global gene expression pattern of the *lvrA/B* and *lvrB* mutant strains by comparing their transcriptome profile to that of the WT *Leptospira*, all grown under standard *in vitro* conditions (30°C in EMJH). Principal Component Analysis (PCA) showed no significant variations between replicates of these RNA seq data (Figure S1). Differential gene expression analyses revealed significant changes in the transcription of 324 genes (~7.5% of the genome) in the *lvrA/B* mutant, 212 genes (~4.9%) in the *lvrB* mutant, and 314 genes (~7.2%) in the *lvrA/B* n *lvrB* set (Figure 3A). Remarkably, 540 genes were derepressed in both *lvr* mutant strains, in comparison to 310 downregulated genes. To validate these gene expression data, we performed quantitative RT-PCR (qRT-PCR) for 17 randomly selected genes from the panel of differentially expressed genes (log ≥ 2-fold; adjusted *p*-value ≤ 0.05). There was a strong agreement between our RNA-Seq and qRT-PCR datasets with a correlation coefficient (*R*^2^) of 0.9119 across the entire panel (Figure 3B).

We employed hierarchical clustering of *lvr* gene expression profiles to uncover biologically relevant expression signatures (Figure 3C). The effect of *lvr* gene inactivation on transcript levels indicated a positive role for Lvr in clusters 1, 4, and 5, and a negative role in clusters 2 and 3 (Figure 3D). To evaluate the potential biological role of the differentially expressed genes we assessed the cluster of orthologous genes (COG) classification system (Figure 3E, Tables S5A–F). This analysis identified the distribution of differentially expressed genes in all 20 COG categories (Figure 3E, Table S6). We observed a significantly increased expression of genes involved in translation (27.6%) and energy production (15.2%), which were distributed in clusters 1 and 5 (Figure 3D). Interestingly, disruption of the Lvr dyad affected 11 distinct operons (47 genes) corresponding to the protein translation machinery (Table S7). This pattern resembles the complex regulation pattern of a cyanobacterial global regulator, PipX, involving 16 genes linked to translation (Espinosa et al., 2014). Additionally, there was an increased expression of genes belonging to signaling and regulation categories (transduction, transcription), primary metabolic processes (lipid transport, nucleotide transport) and metabolic adaptation (cell motility, post-translational modification, and defense mechanisms) in clusters 1, 4, and 5. Genes of unknown function were distributed among all five clusters with relative abundance in downregulated clusters 2 (42.6%) and 3 (57.5%) (Figures 3D,E).

Taken together, we discovered by global expression analysis that Lvr signaling system modifies the expression of ~15% chromosomal genes in *Leptospira* (Figure 3E). This global regulatory role is reminiscent of the *Streptococcus pyogenes* TCS CsrRS (CovRS), which influences the transcription of 15% of its chromosomal genes (Graham et al., 2002).

### LvrAB governs virulence in *Leptospira*

**Figure 4:**
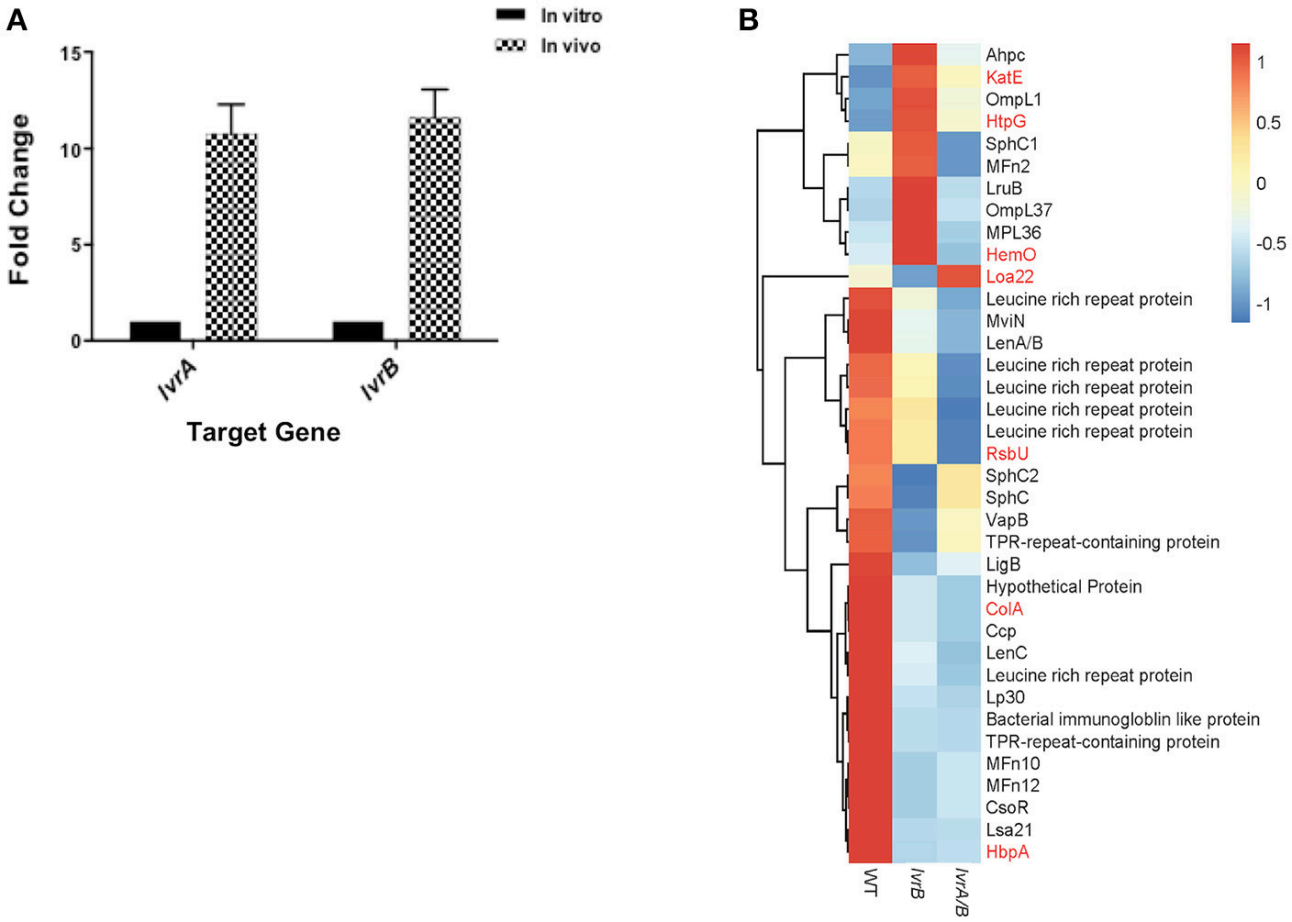
Lvr dyad governs leptospiral virulence. **(A)** *In vivo* expression of *lvr* genes determined by RT-qPCR. Relative expression of the target *lvr* genes was studied by quantifying transcripts in sample collected from blood of hamsters (*n* = 2) at 3 days post-infection intraperitoneally with *L. interrogans* serovar Manilae WT at a dose of 10^8^ *leptospires*. Transcripts of *in vitro* cultures were obtained from a late-log phase culture of *L. interrogans* serovar Manilae WT incubated in EMJH at 30°C. *In vivo* results represent the expression levels of *lvr* genes in comparison to *in vitro* conditions and normalized to *flaB* gene expression. Results are the average of two independent assays and the error bars indicate ±1 SD. **(B)** Categorization of differentially expressed virulence-related genes (*P* < 0.05) in *lvrA/B* (M1529) and *lvrB* (M1419) mutants into genetically characterized (red) genes and putative (black) genes.

Virulence attenuation of *lvr* mutants allowed us to hypothesize that the Lvr system regulates virulence genes in *Leptospira*. *In vivo* assessment of *lvr* gene expression indicated that both *lvrA* and *lvrB* genes were upregulated (9-fold and 12-fold, respectively) in hamster blood, 3 days post-infection via intraperitoneal route with a lethal dose of *Leptospira* WT (Figure 4A). This data is consistent with the direct role of the Lvr system in the infectious process of *Leptospira*.

Furthermore, in our global expression analyses proven and putative virulence genes were differentially regulated in *lvr* mutants. *Leptospira* genes encoding for characterized virulence factors such as collagenase (*colA*), serine phosphatase (*rsbU*) and *h*emin-*b*inding *p*rotein A (*hbpA*) (Asuthkar et al., 2007; Eshghi et al., 2014; Kassegne et al., 2014), were repressed in the *lvr* mutants compared to the wild type strain (Figure 4B). Genes encoding for extracellular matrix proteins with a proposed role in pathogen-host interactions (*ompL1, mfn2, lruB, mpl36, lenA/B, lenC, lp30, mfn10, mfn12, lsa21*; Pinne et al., 2012; Vieira et al., 2014) and for those implicated in host adaptation (*sphC, sphC1, sphC2, mviN*; Caimano et al., 2014) were repressed in *lvr* mutants (Figure 4B). Putative virulence-related genes were also found to be repressed particularly those encoding leucine rich repeats (*lrr*) (Miras et al., 2015), tetratricopeptide repeats (*tpr*) (Cerveny et al., 2013), bacterial immunoglobulin (*mviN*) (Caimano et al., 2014), and copper homeostasis protein (*csoR*) (Liu et al., 2007) (Figure 4B). Taken together, these results indicate that the Lvr system has a major role in mediating virulence regulation through a complex network of genes that ultimately affect the pathogenic potential of *Leptospira*.

### Lvr regulates cell motility

**Figure 5:**
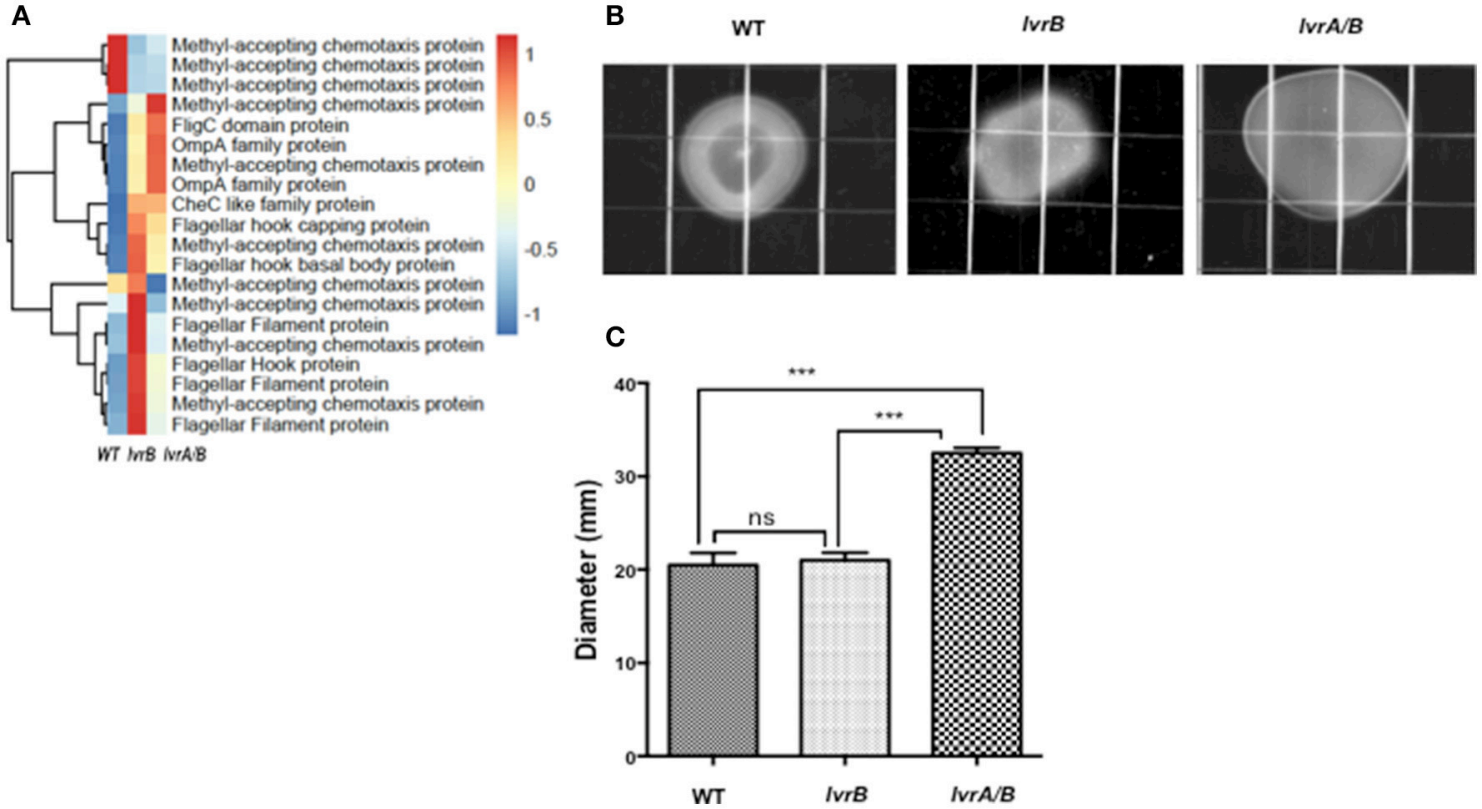
Lvr dyad regulates motility of *Leptospira* spp. **(A)** Hierarchical clustering heatmap representing the normalized expression levels of indicated motility genes in *Leptospira* Manilae L495 wild type, *lvrA/B* (M1529) and *lvrB* (M1419) mutants. **(B)** EMJH plates (0.5% agar) inoculated with 10^5^ cells of *Leptospira interrogans* Manilae L495 wild type strain or *lvrA/B* (M1529) and *lvrB* (M1419) mutants. Plates were incubated at 30°C and colony diameter was measured on 14th day. A representative plate from one of the three experiments is shown. Images were captured by Chemidoc XRS system (BioRad) **(C)** Graphical representation of colony diameter for *Leptospira interrogans* Manilae L495 wild type, *lvrA/B* (M1529) and *lvrB* (M1419) mutants measured after 17 days of incubation on 0.5% semisolid media. Points are plotted at the mean of three biological replicates and error bars indicate ±1 SD. ^***^P ≤ 0.001; ns, not significant.

Interestingly, there was an increased expression of cell motility related genes in both *lvrA/B* (10.37%) and *lvrB* (12.26%) mutants. These genes include those with predicted roles in chemotaxis, as well as flagellar apparatus-related transcripts (Figure 5A). Upon testing of the motility pattern in semisolid media, the *lvrA/B* mutant exhibited an increased spreading phenotype (32.5 mm ± 1) compared to the wild-type strain (20.5 mm ± 1). However, the motility pattern exhibited by the *lvrB* mutant (21 mm ± 0.77) resembled that of the wild-type *Leptospira* (Figures 5B,C). That the *lvrA/B* mutant displayed a distinct phenotype, and not the *lvrB* mutant, supports the most likely hypothesis that LvrA can operate independent of LvrB.

Taken together, the Lvr signaling system seems important to reprogram leptospiral motility. However, the regulation of motility by Lvr is intriguing and warrants further investigation because of the complexity of the flagellar apparatus in *Leptospira* and the lack of understanding of its molecular mechanisms (Wunder et al., 2016).

### Evolution of the LvrA/B system

**Figure 6:**
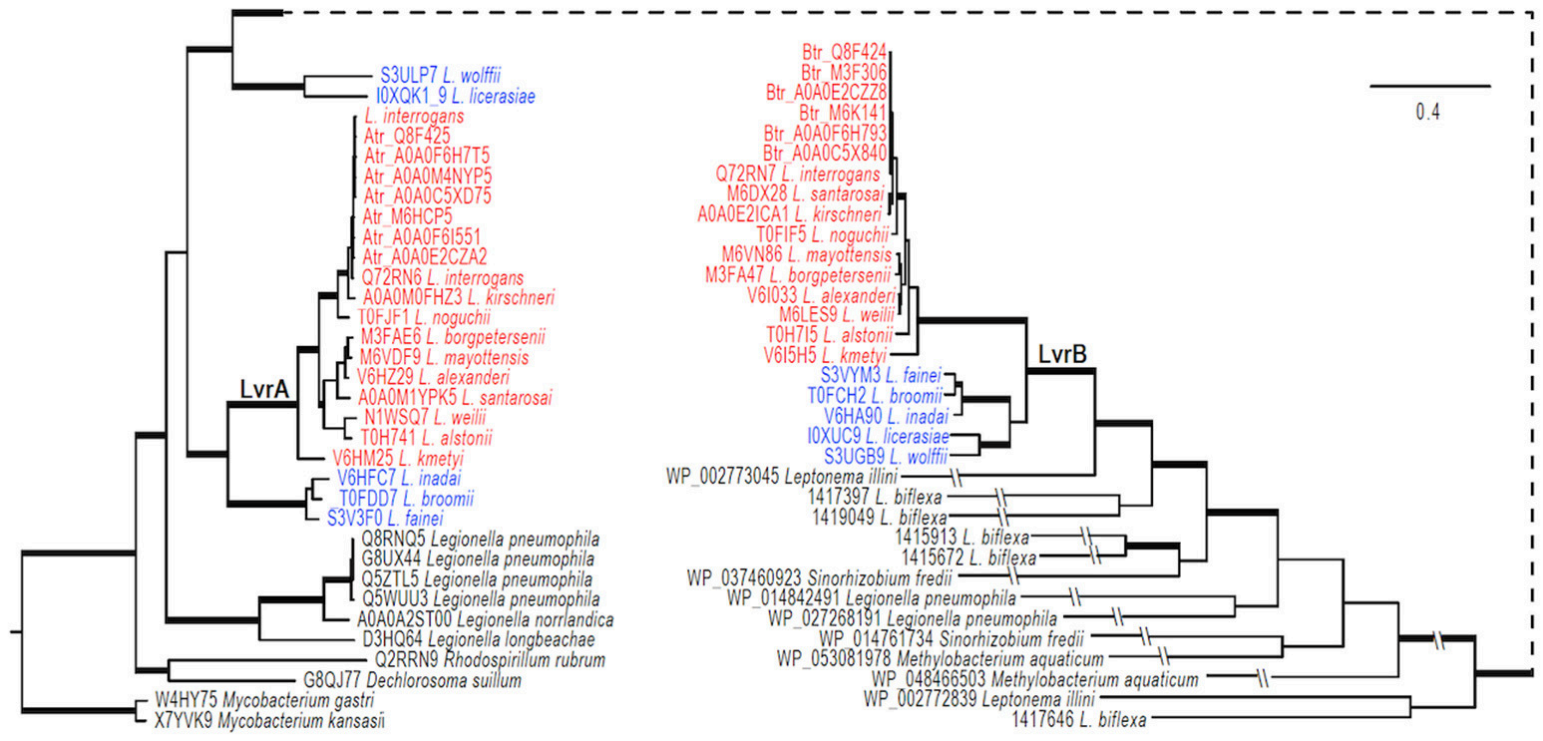
Evolution of LvrA and LvrB. Phylogenetic relationships of *lvr* genes in pathogenic *Leptospira* (indicated in red) with intermediate *Leptospira* (indicated in blue) and related two-component systems in sampled bacteria were inferred from an amino acid alignment using Bayesian approaches with models averaged parameter sets of rate matrix. The trees were rooted with sequences from two *Mycobacterium* species. The majority-rule consensus of 8001 MCMCMC-sampled trees with averaged branch length is present, and branches with strong support (BPP > 0.98) are in boldface. Bar indicates the substitutions per amino acid site. The tree is broken at a node for a better presentation, and a dashed line is used to link the node. Exceedingly long branches are foreshortened, as indicated with the symbol -//-.

The genomic region comprising the *lvrA* and *lvrB* genes was found only in pathogenic *Leptospira* species, suggesting that these genes could have been acquired through lateral gene transfer. To test this hypothesis, phylogenetic analyses were performed on an alignment of *lvr* gene orthologs in pathogenic *Leptospira* species (with >80% identity), also including putative hybrid HK/hybrid RR hits in intermediate species (with ~50% identity) and saprophytic species (with <30% identity). PanOCT ortholog clustering identified the hits from intermediate *Leptospira* species as divergent paralogs with a different gene neighborhood than in pathogens (Fouts et al., 2016). Our search for *lvr* orthologs in non-*Leptospira* genomes revealed closest hits (with ~40% identity) in gram-positive *Mycobacterium* spp., beta proteobacteria *Dechlorosoma* spp., and gamma proteobacteria *Legionella* spp., suggesting that these genes are of ancient origin. We identified six *lvr* orthologs in *Legionella* (with 40% identity and these orthologs clustered with *lvrA* of *Leptospira* (Figure 6). *Lvr* and its gene family are absent from other spirochetes such as *Treponema* and *Borrelia*. These results are consistent with the notion that *Leptospira* acquired a progenitor of *lvr* through lateral gene transfer.

Our phylogenetic analyses indicated that genes specifying the Lvr signaling system experienced at least one gene duplication event after the acquisition of the progenitor *lvr* genes (Figure 6). This is because distinct clades were observed for intermediate vs. pathogenic *Leptospira* species. Furthermore, PanOCT ortholog clustering of paralogs from intermediate species suggests lineage-specific duplication (Figure 6). For instance, search for a PAS domain-encoding *lvrA* did not yield any hits in the saprophytic species *L. biflexa*, suggesting a specialized role for this domain in expanding the signaling capabilities of pathogenic *Leptospira*. Likewise, putative RRs identified from saprophytic *Leptospira* species showed a distant evolutionary origin with *lvrB* orthologs of intermediate species, but not with pathogenic *Leptospira*. This study supported the proposition that duplicated genes relevant to virulence would become fixed in pathogenic lineages, while they could be lost in saprophytic lineages (Powell et al., 2008). Therefore, Lvr proteins constituting regulatory networks in pathogenic *Leptospira* spp. most likely provides a selective advantage related to their infectious ability and lifestyle diversity.

### Conceptual model for Lvr regulatory system

**Figure 7:**
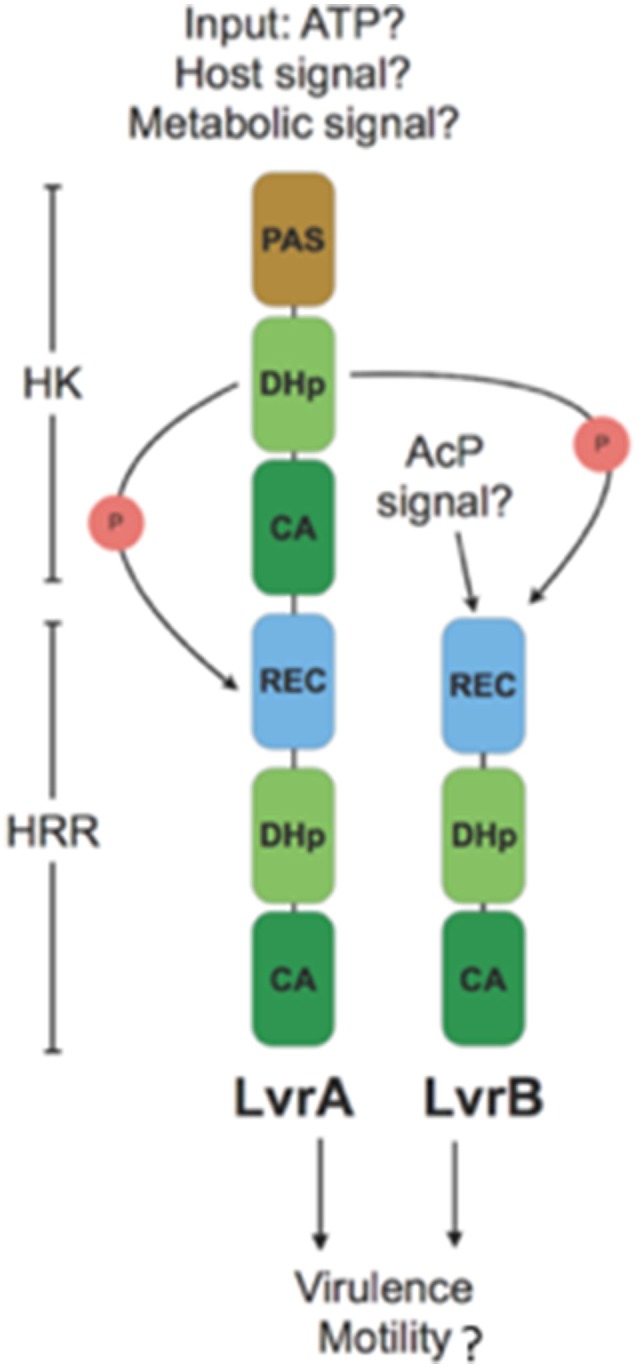
Model for branched signaling pathway of Lvr hybrid two-component system: An unknown input signal modulates the autokinase activity of N-terminal HK module in LvrA. Upon switching to a kinase-on state, there would be phosphotransfer to its downstream HRR module, as well as to LvrB in a branched pathway. Alternatively, LvrB can be activated by a small molecule signal such as AcP (Acetyl Phosphate). After phosphotransfer events, Lvr proteins influence the expression of virulence and motility genes either by activation of putative downstream RRs or by protein-protein interactions.

Activation of the Lvr TCS might depend upon signal sensing by the single sensory PAS domain found in LvrA protein (Figure 7). The fact that *lvr* genes are induced *in vivo* after host infection (Figure 4A), strongly suggests that a specific input signal(s), yet to be determined, is present within the host environment. In cytoplasmic HKs, PAS domains may mediate protein-protein interactions, or could be involved in binding co-factors through which sensing of oxygen, light or yet cellular redox state have been demonstrated (Henry and Crosson, 2011). Distortions in the PAS central β-sheet due to ligand binding can cause quaternary changes, which are transmitted along the HK dimer interface toward the kinase transmitter domain and eventually modulate the output HK activity (Cheung and Hendrickson, 2010). In a signal-dependent way, the PAS domain of LvrA likely modulates the autokinase activity of its first HK module, according to a scenario found in canonical PAS-containing HKs (Cheung and Hendrickson, 2010). To date, only few cytosolic (soluble) HKs have been reported including the NtrBC protein in enteric bacteria, KinA in *Bacillus subtilis*, HoxJA in *Ralstonia eutropha*, TodS in *P. putida* and ThkA in *T. maritima*. PAS domains of these HKs sense environmental stimuli were found to have direct access to the cell by diffusion or transmission (Reitzer, 1996; Lenz and Friedrich, 1998; Phillips and Strauch, 2002; Busch et al., 2007; Gao and Stock, 2009; Yamada et al., 2009). In pathogenic bacteria, the only examples include DosS and DosT in *M. tuberculosis*; each of these harbors PAS domains that respond to low O_2_ (Kumar et al., 2007). Alternatively, small molecules such as acetyl phosphate might function *in vivo* as a signal under certain metabolic conditions, by donating its phosphoryl group to certain response regulators independent of HK (Wolfe, 2010). Given the cytosolic location of the Lvr system, molecules like acetyl phosphate could serve as a potential signal, directly activating LvrB protein devoid of a PAS domain but with an N-terminal REC domain (Figure 7).

LvrA autophosphorylates on His218, and preferentially transfers the phosphate group to its own conserved aspartate downstream (Asp524). Alternatively, LvrA can also phosphotransfer to Asp56 on LvrB as well (Figure 7). This activation mode of LvrB, lacking a sensor domain of its own, is comparable to *Sphingomonas melonis* PakF, which is phosphorylated by the HK KipF during stress response (Kaczmarczyk et al., 2015). Phosphotransfer to reactive aspartate residues within the HRR regions of both LvrA and LvrB, likely controls the kinase activities of their effector HK domains and in fine modulating downstream RRs. Given the global transcriptional regulation effect found in *lvr* mutant strains (Figure 3A), we posit that RRs with direct transcriptional regulation capacity are likely to work downstream of Lvr. This is also supported by the fact that both LvrA and LvrB lack DNA-binding effector domains within their HRR modules.

We identified eight putative RRs harboring DNA-binding effector domains in the *L. interrogans* genome (Table S8). All of these are located in the genome adjacent to an HK gene, hence likely functioning as cognate partners in TCSs. Lvr signaling system is thus expected to interact with one or more of these TCSs, giving rise to an inherently branched pathway, ultimately interfering with those TCSs that control DNA transcription via a more complex network of phosphotransfer events. The fact that LvrA and LvrB are unusually abundant proteins, compared to the typical concentration ranges found for signaling components in the cell (Malmström et al., 2009), and that they are overexpressed during infection (Figure 4A), is consistent with their potential ability to cross-talk. This cross-talk could be achieved by overcoming specific paired interactions among cognate HKs and RRs, ultimately subverting the activation of one or more DNA-binding RRs.

Examples of branched signaling in bacteria have been described in a number of different pathways, including some mediated by HRRs (Garzon and Parkinson, 1996; Kaczmarczyk et al., 2015). The topology of branching in the case of Lvr is expected to correspond to a divergent cascade, with signal triggered information flow going from LvrA to LvrB to downstream effector partners or it could be directly from LvrA to other effectors. Moreover, we cannot exclude the possibility that LvrB could also be activated by alternative upstream kinases other than LvrA itself. This divergent and branched signaling flow is anticipated to allow for a particularly diverse set of adaptive responses that *Leptospira* mounts, fine-tuned to the extremely varied range of growth niches in which these spirochetes are able to live in (Figure 7). In our transcriptome analysis of *lvr* mutants, we observed differentially expressed genes (13%) belonging to transcriptional and signal transduction categories (Figure S2). A rich regulatory network can thus be envisaged, similar to the one controlled by the BvgA/BvgS TCS of *Bordetella pertussis*, where a multistep His-Asp-His phosphorelay occurs between different kinase domains prior to phosphorylation of the BvgA response regulator (Uhl and Miller, 1996). Therefore we cannot exclude the possibility that LvrAB directly interact with DNA-binding proteins, thus exerting the global regulatory effect (Gao et al., 2007). Further studies should be conducted to determine if the transcriptional differences observed between the WT and *lvr* mutant strains are due to interaction of LvrA/B proteins with collateral regulatory networks.

In summary, the identification of Lvr, a TCS that controls virulence and motility in pathogenic *Leptospira* unveiled the existence of a complex signaling network in this genus. To the best of our knowledge, this is the first report of a virulence-associated two-component system in this important zoonotic pathogen. The evidence of global transcriptional regulation by Lvr presented in this study allows us to speculate its role in dynamic modulation of metabolic activities and expression of virulence determinants. These new findings related to Lvr will provide us with a defined framework to identify “classical” pathogenic factors (e.g. toxins, adhesins, secretion apparatuses, etc.) under its coordinated regulation through which *Leptospira* can reprogram and adapt to the host.

## Author contributions

HA, EW, AM, EG, AB, and AK: Conceived and designed the experiments; HA, EW, AM, and VB: Performed the experiments; Data Analysis and Interpretation: HA, EW, AM, SM, ZW, LS, PD, FL, JT, EG, AB, and AK; PD, GM, BA, FL, JT, MP, AB, and AK: Contributed reagents, materials, analysis tools; HA: Drafted the manuscript; EW, AM, SM, ZW, LS, PD, GM, BA, FL, JT, EG, MP, AB, and AK: Revised the paper.

### Conflict of interest statement

The authors declare that the research was conducted in the absence of any commercial or financial relationships that could be construed as a potential conflict of interest.
