# Supplementary material for: Lvr, a Signaling System That Controls Global Gene Regulation and Virulence in Pathogenic Leptospira
Source: Front Cell Infect Microbiol. 2018 Feb 23;8:45. doi: 10.3389/fcimb.2018.00045 (PMC5863495; doi:10.3389/fcimb.2018.00045)
Supplement: Supplementary file 1 [file Table1.DOC]

**Table S1: Bacterial Strains**

| **Strain** | **Description^1^** | **Source** |
| --- | --- | --- |
| *Leptospira interrogans* serovar Manilae strain L495 | Wild type strain (WT) |  |
| *lvrA/B* (M1529) | Transposon insertion in *lic11709* (LMANv2_670019: Hybrid HK protein) | This study |
| *lvrA/B* II (M1529 II) | Transposon insertion in *lic11709* (LMANv2_670019: Hybrid HK protein) | This study |
| *lvrB* (M1419) | Transposon insertion in intergenic region between *lic11708* and *lic11709* (LMANv2_670019: Hybrid HK protein  LMANv2_670020: Hybrid RR protein) | This study |
| *lic13192* (M480) | Transposon insertion in *lic13192* (LMANv2_690010: Hybrid HK/RR protein) | This study |
| *lic13087* (M854) | Transposon insertion in *lic13087* (LMANv2_700002: HK protein) | This study |

^1^Gene ID’s corresponding to both *L. interrogans* *serovar Copenhageni* Fiocruz L1-130 and *L. interrogans serovar Manilae* are indicated

Abbreviations, HK: Histidine Kinase; RR: Response Regulator
