# Supplementary material for: Lvr, a Signaling System That Controls Global Gene Regulation and Virulence in Pathogenic Leptospira
Source: Front Cell Infect Microbiol. 2018 Feb 23;8:45. doi: 10.3389/fcimb.2018.00045 (PMC5863495; doi:10.3389/fcimb.2018.00045)
Supplement: Supplementary file 3 [file Table3.DOC]

**Table S3: Summary of *in vivo* experiments via conjunctival route in hamster model of infection.**

| **Strain** | **Expt. No. *(n^a^)*** | **Conjunctival Dose** | **% Mortality** | **p-Value (Fisher Test)** | **Days for death** | **p-Value (Mantel-Cox)** |
| --- | --- | --- | --- | --- | --- | --- |
| WT | 1 (8) | 10 ^8^ | 100 | - | 8,9,9,9,9,9,9,9 | - |
|  | 2 (6) | 10 ^7^ | 100 | - | 7,8,8,9,12,12 | - |
|  | 3 (8) | 10 ^7^ | 100 | - | 8,9,9,10,10,10,10,11 | - |
|  | 4 (8) | 10 ^7^ | 100 | - | 8,8,8,9,9,10,10,10 | - |
|  | 5 (10) | 10 ^7^ | 100 | - | 8,8,9,9,10,10,10,10,11,13 | - |
|  | 6 (6) | 5 x 10^6^ | 83 | - | 9,9,16,17,18 | - |
| *lvrA/B* | 1 (8) | 10 ^8^ | 50 | 0.0769 | 10,10,10,11 | 0.0001 |
|  | 2 (6) | 10 ^7^ | 33 | 0.030 | 9,9 | 0.0189 |
|  | 3 (8) | 10 ^7^ | 0 | 0.0002 | - | 0.0001 |
|  | 4 (8) | 10 ^7^ | 38 | 0.012 | 10,10,11 | 0.0008 |
|  | 6 (6) | 5 x 10^6^ | 0 | 0.007 | - | 0.004 |
| *lvrB* | 1 (8) | 10 ^8^ | 0 | 0.0002 | - | 0.0001 |
|  | 2 (6) | 10 ^7^ | 0 | 0.002 | - | 0.0007 |
|  | 3 (8) | 10 ^7^ | 13 | 0.001 | 13 | 0.0001 |
|  | 4 (8) | 10 ^7^ | 38 | 0.012 | 10,10,11 | 0.0008 |
|  | 6 (6) | 5 x 10^6^ | 0 | 0.007 | - | 0.004 |
| *lvrA/B II* | 4 (8) | 10 ^7^ | 50 | 0.038 | 9,9,9,12 | 0.013 |
|  | 5 (10) | 10 ^7^ | 10 | 0.0001 | 14 | 0.0001 |
| *lic13192* | 2 (6) | 10 ^7^ | 67 | 0.45 | 7,8,8,9 | 0.46 |
|  | 6 (6) | 5 x 10^6^ | 67 | 0.5 | 8,8,15,16 | 0.96 |
| *lic13087* | 2 (6) | 10 ^7^ | 83 | 1 | 7,8,8,8,9 | 0.74 |

^a^ n represents number of hamsters employed in that particular experiment.
