# Supplementary material for: Lvr, a Signaling System That Controls Global Gene Regulation and Virulence in Pathogenic Leptospira
Source: Front Cell Infect Microbiol. 2018 Feb 23;8:45. doi: 10.3389/fcimb.2018.00045 (PMC5863495; doi:10.3389/fcimb.2018.00045)
Supplement: Supplementary file 4 [file Table4.DOC]

**Table: S4 Model of dose-response relationship for conjunctival infection of**

**hamsters with Leptospira strains.**

Data represents dose effect modeled as power law, hazard proportional to dose^a^, with 95% confidence interval for a = 0.352 +/- 0.562.

| ***Leptospira* strain** | **Beta** | **Sebeta** | **z** | **P** |
| --- | --- | --- | --- | --- |
| *lvrA/B* | -2.593919 | 0.400640 | -6.474434 | 0.000000 |
| *lvrB* | -3.548376 | 0.552394 | -6.423630 | 0.000000 |
| *lvrA/B II* | -2.463665 | 0.515715 | -4.777187 | 0.000002 |
| *lic13192* | 0.732820 | 0.626373 | -1.169943 | 0.242024 |
| *lic13087* | 0.016972 | 0.584078 | 0.029058 | 0.976819 |

D
