# Supplementary material for: Lvr, a Signaling System That Controls Global Gene Regulation and Virulence in Pathogenic Leptospira
Source: Front Cell Infect Microbiol. 2018 Feb 23;8:45. doi: 10.3389/fcimb.2018.00045 (PMC5863495; doi:10.3389/fcimb.2018.00045)
Supplement: Supplementary file 6 [file Table6.DOC]

| **COG Category (n)^a^** | **% Distribution of differentially expressed genes** | | | | |
| --- | --- | --- | --- | --- | --- |
|  | **Cluster 1** | **Cluster 2** | **Cluster 3** | **Cluster 4** | **Cluster 5** |
| C (138) | 15.94 | 1.45 | 0.72 | 0.72 | 15.94 |
| D (54) | 3.70 | 5.56 | NA | NA | 16.67 |
| E (209) | 2.87 | 1.91 | NA | 2.87 | 1.91 |
| G (137) | 3.65 | 1.46 | 0.73 | 1.46 | 3.65 |
| H (107) | 0.93 | 3.74 | 0.93 | 1.87 | 2.80 |
| I (114) | 11.21 | 1.87 | 0.93 | 4.67 | 1.87 |
| J (159) | 6.29 | NA | 0.63 | 4.40 | 30.82 |
| K (142) | 4.93 | 4.93 | NA | 8.45 | 5.63 |
| L (165) | 3.64 | 3.64 | 2.42 | 1.82 | 4.85 |
| M (231) | 4.76 | 0.87 | 0.43 | 4.33 | 3.90 |
| N (106) | 10.38 | 0.94 | 1.89 | 0.94 | 4.72 |
| UC (1835) | 4.85 | 8.12 | 4.03 | 1.58 | 2.67 |
| O (118) | 13.56 | NA | NA | 4.24 | 5.93 |
| P (133) | 3.76 | 2.26 | 0.75 | 4.51 | 2.26 |
| Q (62) | 6.45 | 4.84 | 1.61 | 3.23 | 3.23 |
| R (455) | 2.64 | 3.08 | 0.88 | 3.08 | 4.18 |
| S (216) | 6.94 | 6.94 | 1.39 | 1.85 | 3.24 |
| T (240) | 5.42 | 2.08 | 1.67 | 4.17 | 5.83 |
| U (71) | 7.04 | 4.23 | 1.41 | 8.45 | 7.04 |
| V (46) | 2.17 | 2.17 | 2.17 | 6.52 | 4.35 |
| F (55) | NA | 1.82 | NA | 7.27 | 3.64 |
| Z (2) | NA | 50.00 | NA | NA | NA |

**Table S6: Percentage distribution of differentially expressed genes across clusters.**

^a^ Total number of genes in corresponding COG category in *L. interrogans* Manilae genome
