# Supplementary material for: Lvr, a Signaling System That Controls Global Gene Regulation and Virulence in Pathogenic Leptospira
Source: Front Cell Infect Microbiol. 2018 Feb 23;8:45. doi: 10.3389/fcimb.2018.00045 (PMC5863495; doi:10.3389/fcimb.2018.00045)
Supplement: Supplementary file 8 [file Table8.DOC]

**Table S8: List of putative response regulators harboring DNA binding domains in the genome of *Leptospira interrogans* Manilae L495.**

| **Gene ID** | **Annotation** |
| --- | --- |
| LMANv2_530024  LMANv2_590040  LMANv2_290023  LMANv2_240098  LMANv2_700001  LMANv2_330031  LMANv2_90110  LMANv2_90069 | KdpE  NtrX  CreB  Hypothetical protein  Hypothetical protein.  PhoP  Hypothetical protein  Hypothetical protein |
