# Supplementary material for: Lvr, a Signaling System That Controls Global Gene Regulation and Virulence in Pathogenic Leptospira
Source: Front Cell Infect Microbiol. 2018 Feb 23;8:45. doi: 10.3389/fcimb.2018.00045 (PMC5863495; doi:10.3389/fcimb.2018.00045)
Supplement: Supplementary file 9 [file Image1.PDF]

Figure S1: Principal Component Analysis for RNA-Seq data.

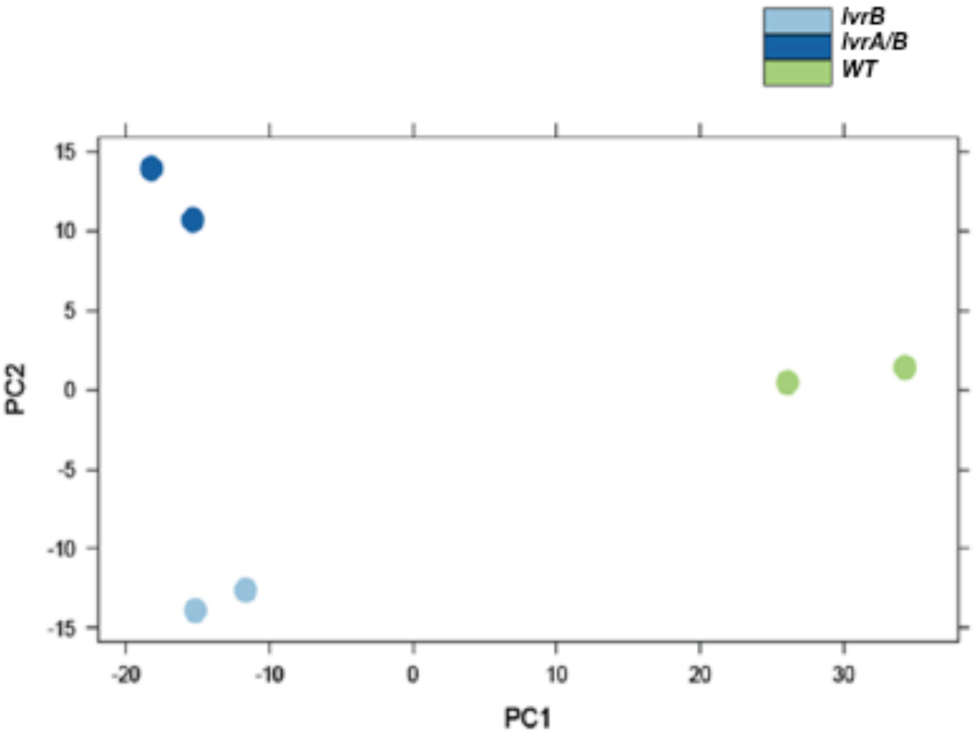

First two principal components for RNA Seq data have been plotted.
