# Supplementary figures and images for: Lvr, a Signaling System That Controls Global Gene Regulation and Virulence in Pathogenic Leptospira
Source: Front Cell Infect Microbiol. 2018 Feb 23;8:45. doi: 10.3389/fcimb.2018.00045 (PMC5863495; doi:10.3389/fcimb.2018.00045)

Figure S2: Heatmap of differentially regulated signal transduction genes in *lvr* mutants

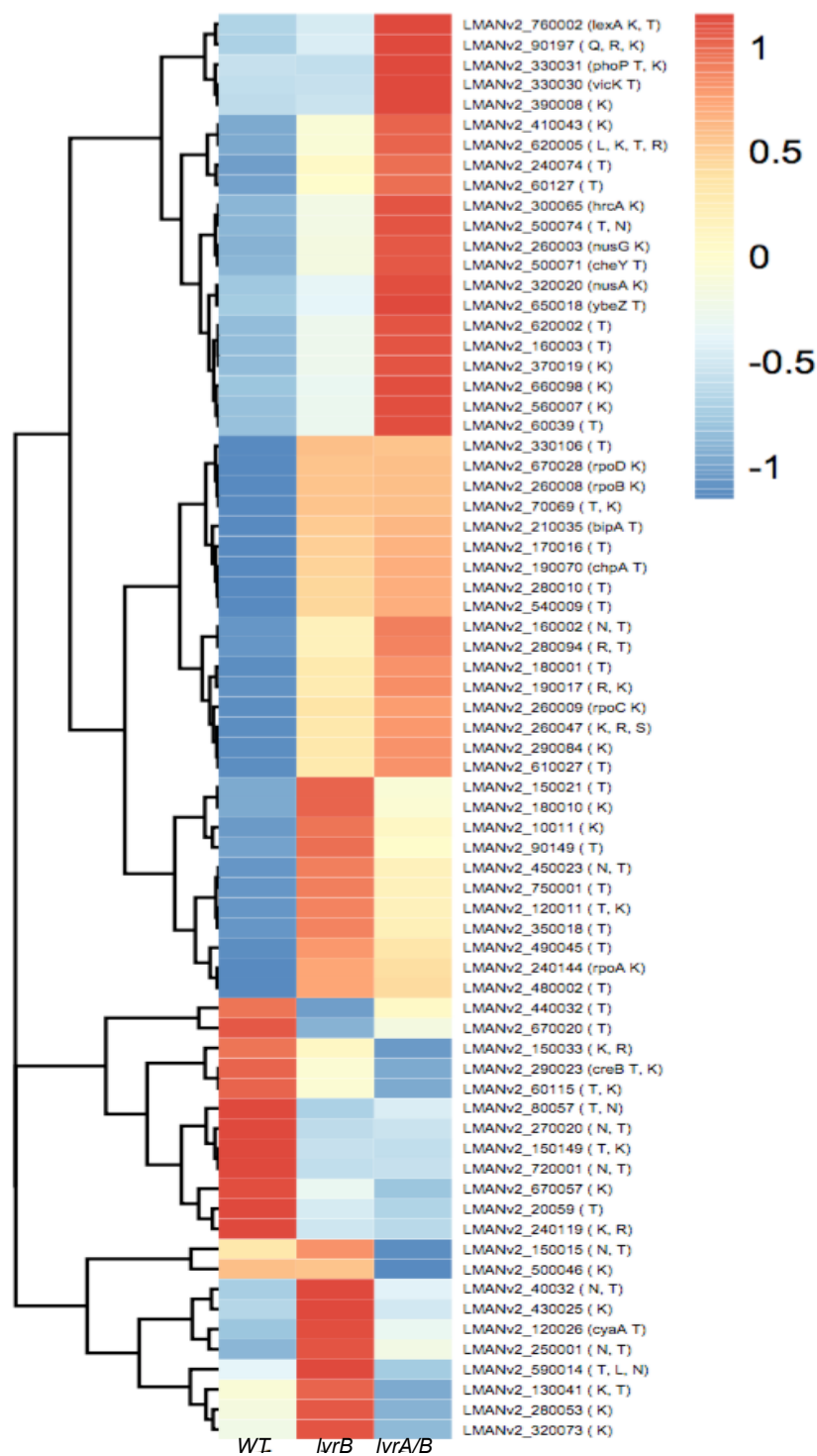

Supplement: Supplementary file 10 [file Image2.PDF]
